# Poor sleep in middle-aged women is not associated with menopause *per se*

**DOI:** 10.1590/1414-431X20154718

**Published:** 2015-11-17

**Authors:** M.F. Tao, D.M. Sun, H.F. Shao, C.B. Li, Y.C. Teng

**Affiliations:** Department of Obstetrics and Gynecology, Shanghai Jiao Tong University Affiliated to the Sixth People's Hospital, Shanghai, China

**Keywords:** Kupperman Index, Menopause, Menopause Rating Scale, Pittsburgh Sleep Quality Index, Sleep disorder, Quality of life

## Abstract

Whether sleep problems of menopausal women are associated with vasomotor symptoms and/or changes in estrogen levels associated with menopause or age-related changes in sleep architecture is unclear. This study aimed to determine if poor sleep in middle-aged women is correlated with menopause. This study recruited women seeking care for the first time at the menopause outpatient department of our hospital. Inclusion criteria were an age ≥40 years, not taking any medications for menopausal symptoms, and no sleeping problems or depression. Patients were assessed with the Pittsburgh Sleep Quality Index (PSQI), modified Kupperman Index (KI), and Menopause Rating Scale (MRS). A PSQI score of <7 indicated no sleep disorder and ≥7 indicated a sleep disorder. Blood specimens were analyzed for follicle-stimulating hormone and estradiol levels. A total of 244 women were included in the study; 103 (42.2%) were identified as having a sleep disorder and 141 as not having one. In addition, 156 (64%) women were postmenopausal and 88 (36%) were not menopausal. Follicle-stimulating hormone and estradiol levels were similar between the groups. Patients with a sleep disorder had a significantly higher total modified KI score and total MRS score (both, P<0.001) compared with those without a sleep disorder. Correlations of the PSQI total score with the KI and MRS were similar in menopausal and non-menopausal women. These results do not support that menopause *per se* specifically contributes to sleep problems.

## Introduction

Sleep complaints are common among the general population, with approximately 35% of individuals having problems falling asleep, staying asleep, awakening early, or not feeling refreshed after sleep (1). Conditions associated with difficultly sleeping include old age, stress, alcohol and drug abuse, depression, lower educational and socioeconomic status, disease, and female sex (2). Sleep disorders have a significant effect on quality of life, as well as societal and economic effects with respect to lost productivity, accidents, and health care costs (3). Insomnia is defined as difficulty initiating or maintaining sleep, accompanied by irritability or fatigue during wakefulness (4). Insomnia is estimated to affect up to 30% of the general population and women are almost twice as likely as men to develop it (5,6). Sleep difficulties among women also increase during the peri- and postmenopausal periods.

Menopause is characterized by a marked decrease in the production of female sex hormones, most specifically estrogen, which results in vasomotor symptoms, such as hot flashes and night sweats (7). The prevalence of insomnia has been shown to increase in women at the age of transition from pre- to postmenopausal stages, and menopausal women are more than three times as likely to have a sleep disorder as those who are premenopausal (8). Some studies has shown that insomnia is more closely associated with psychological than somatic symptoms of menopause (9). Despite considerable research, whether sleep problems are associated with hormonal status, vasomotor symptoms of menopause, or age-related changes in sleep architecture remains unclear (10).

Evaluation and quantification of menopausal symptoms in a standardized manner are important for diagnosis and treatment. Two of the most commonly used instruments are the Kupperman Index (KI) (11) and the Menopause Rating Scale (MRS) (12). Both of these instruments are self-administered questionnaires designed to measure menopausal symptoms, they are widely accepted, and the correlation between the two instruments is strong (13,14). The Pittsburgh Sleep Quality Index (PSQI) is a validated self-administered questionnaire that assesses sleep quality and disturbances. The PSQI can be used to categorize people as good or poor sleepers (i.e., those affected by a sleep disorder) (15).

This study aimed to determine if poor sleep, as evaluated using the PSQI, is correlated with menopause and quality of life, as evaluated with the KI and MRS, in pre- and postmenopausal women.

## Material and Methods

### Participants and testing

Women who were seen for the first time at the menopause outpatient department of the Department of Gynecology, the Sixth Affiliated People's Hospital of Shanghai Jiao Tong University, Shanghai, China, between January 2011 and June 2011 were recruited. The study was approved by the Institutional Review Board of the Sixth Affiliated People's Hospital of Shanghai Jiao Tong University (#2010-06). All of the patients provided written informed consent for participation in the study.

Inclusion criteria were an age ≥40 years, not taking hormone replacement therapy or any other medications for menopausal symptoms, not taking medications for sleeping problems, not taking antidepressants, no severe systemic diseases, and willingness to complete the study questionnaires. Patients with severe diseases, and those who underwent hysterectomy or oophorectomy were excluded. Overweight was not an exclusion criterion.

All of the participants completed a form to provide demographic data, including age, education level, income, marital status, occupation, menopausal status, and disease history. Pre-menopause was defined as patients who still had menstrual flow and postmenopause was defined as no menstrual flow. The length of time since periods ceased was recorded. All of the patients were categorized based on the 2001 Stages of Reproductive Aging Workshop (STRAW) simplified bleeding criteria for early and late menopausal transition (16). At the visit, patients were required to provide a blood sample for determination of follicle-stimulating hormone (FSH) and estradiol (E2) levels. Blood specimens were analyzed according to standard procedures. Participants' height and weight were measured, and their body mass index (BMI, kg/m^2^) was calculated. Systolic blood pressure and diastolic blood pressure were recorded.

All of the participants completed the Chinese versions of the PSQI (15), the MRS (12,17), and the modified KI (14) questionnaires. The PSQI and MRS have been validated in the Chinese language (18,19). The order in which the questionnaires were completed was random. Two experienced interviewers (SHF and LCB) provided all of the surveys, and answered any questions that were raised by the participants.

The PSQI assesses sleep quality and disturbances over a 1-month period (15). This instrument consists of 19 items that are used to generate seven component scores: subjective sleep quality, sleep latency, sleep duration, habitual sleep efficiency, sleep disturbances, use of sleeping medication, and daytime dysfunction. Answers are scored on a 0-3 scale, with 0 indicating no dysfunction and 3 indicating the worst dysfunction. The sum of the scores is considered as the global score. A higher global score indicates a "worse" sleeper. In this study, a PSQI global score <7 was considered to indicate no sleep disorder and a PSQI global score ≥7 was used to indicate a sleep disorder.

The MRS consists of 11 items that are categorized into three subscales: 1) somatovegetative symptoms: sweating/hot flashes, heart discomfort, sleep problems, and joint and muscle problems, 2) psychological symptoms: depressive mood, irritability, anxiety, and physical/mental exhaustion, and 3) urogenital symptoms: sexual problems, bladder problems, and vaginal dryness (12). Severity is scored as none (no points), mild (one point), moderate (two points), severe (three points), and very severe (four points), with a range in total score from 0-44. Scores ranging from 0-4, 5-8, 9-15, and 16+ are typically used to rate menopausal symptoms as none/minimal, mild, moderate, and severe, respectively (20).

The original KI consists of questions regarding 11 items, including sweating/hot flashes, palpitation, vertigo, headache, paresthesia, formication, arthralgia, and myalgia (categorized as somatic symptoms), as well as fatigue, nervousness, and melancholia (categorized as psychological symptoms) (11). The modified KI (14) consists of 13 items. In addition to the 11 items in the original KI, the modified version adds items regarding urogenital symptoms, such as urinary tract infection and sexual complaints. The severity of each symptom is rated on a scale from 0 to 3 for no, slight, moderate, and severe complaints, respectively, and the highest potential score is 63.

### Data analysis

Continuous data are reported as means±SD or medians with interquartile range (IQR, 25th and 75th percentile), depending on normality of data distribution. Categorical data are shown as frequencies and percentages. The differences between women with a sleep disorder (PSQI ≥7) and those without (PSQI <7) were detected by the independent *t*-test or the Wilcoxon rank sum test for continuous data, and by the chi-square test or the Fisher's exact test for categorical variables, as appropriate. The relationships between the PSQI total score and modified KI score, as well as the MRS score, were measured item-by-item using Spearman's rank correlation coefficient for a) total patients (denoted as r_a_), b) patients not in menopause (denoted as r_b_), and c) patients in menopause (denoted as r_c_). The correlation strength was evaluated based on the following scale: very weak (0-0.19), weak (0.20-0.39), moderate (0.40-0.59), strong (0.60-0.79), and very strong (0.80-1.00). Statistical analyses were performed with the SAS software version 9.2 (SAS Institute Inc., USA). A two-tailed value of P<0.05 indicated statistical significance.

## Results

### Patients' characteristics

**Figure 1:**
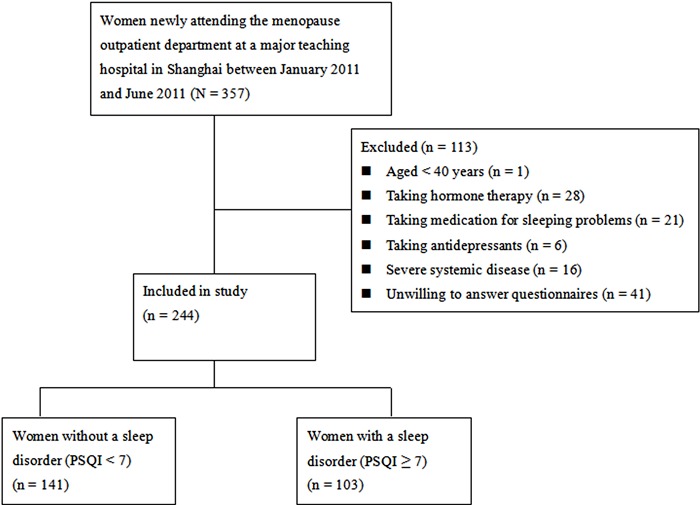
Flow chart of selection of patients. PSQI: Pittsburgh Sleep Quality Index.

A flow diagram of selection of the patients is shown in Figure 1. Of 357 patients screened, a total of 244 women with a mean age of 51.65±4.78 years (range, 40-67 years) were included in this study (Table 1). All of the patients were -2 to +2 based on the 2001 STRAW +10 staging system. A total of 103 (42.2%) women were identified as having a sleep disorder (PSQI ≥7) and 141 were categorized as not having a sleep disorder (PSQI <7). Most of the women (178 subjects, 73.0%) were aged between 45 and 55 years, and 97.5% were married. Approximately half (51.2%) of the subjects were retired. Age, BMI, systolic blood pressure, diastolic blood pressure, marital status, occupational status, educational status, monthly income, age at menarche, and menopausal status were not different between women with a sleep disorder and those without (all P>0.05, Table 1).

**Table:**
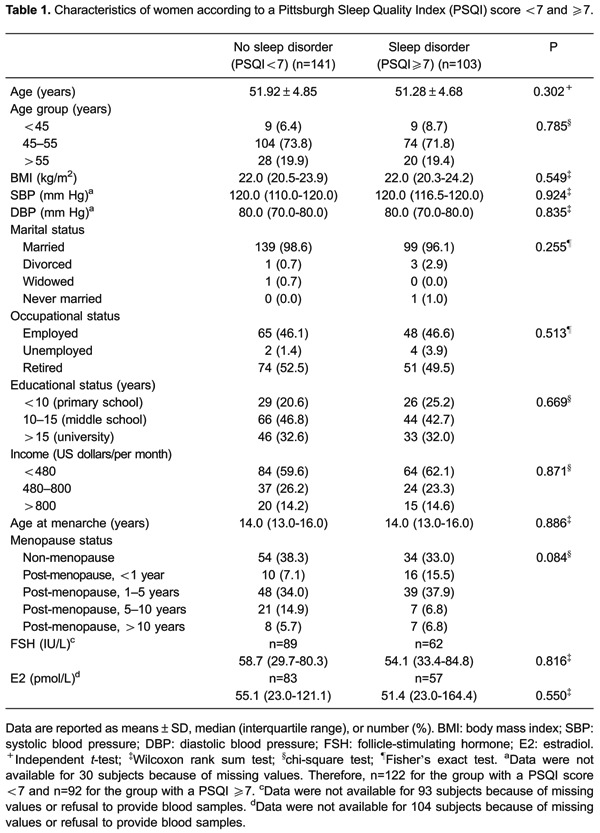

### FSH and E2 levels

The median serum FSH and E2 levels of women without a sleep disorder were 58.7 IU/L (29.7-80.3 IU/L) and 55.1 pmol/L (23.0-121.1 pmol/L), and 54.1 IU/L (33.4-84.8 IU/L) and 51.4 pmol/L (23.0-164.4 pmol/L) for those with a sleep disorder, respectively. No significant differences in serum FSH and E2 levels were found between the two groups (Table 1, both P>0.05).

### Modified KI scores

Comparison of the item-by-item modified KI scores of women with and without a sleep disorder is shown in Table 2. Unsurprisingly, women with a sleep disorder had a significantly higher score for insomnia compared with those without a sleep disorder (4.47±1.29 *vs* 1.35±1.38, P*<*0.001). KI scores for sweating, hot flashes, nervousness, melancholia, vertigo, fatigue, headache, heart palpitations, formication, and sexual complaints were significantly greater in women with a sleep disorder compared with those without a sleep disorder (all P<0.05). Overall, even without the inclusion of insomnia, those with a sleep disorder had a significantly higher total modified KI score compared with those without (23.54±9.05 *vs*17.59±8.24, P<0.001).

**Table:**
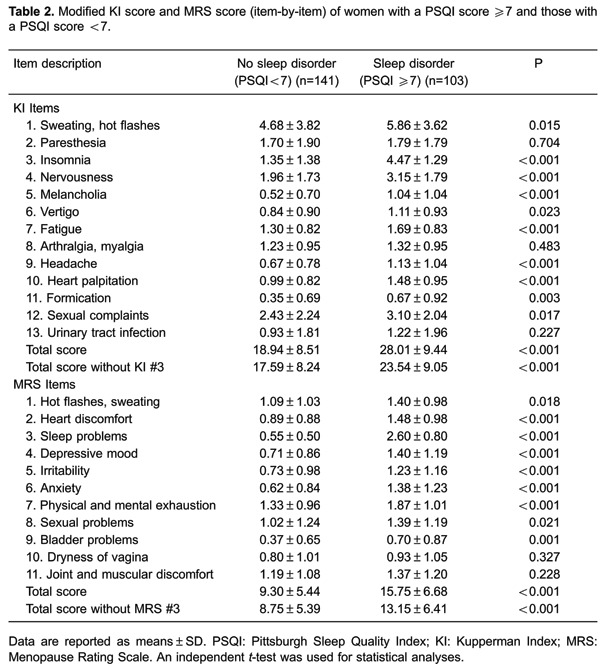

### MRS scores

Comparison of the item-by-item MRS score of women with and without a sleep disorder is shown in Table 2. Similar to the modified KI score, women with a sleep disorder had a significantly higher score for sleeping problems (MRS 3) compared with those without a sleep disorder (2.60±0.80 *vs* 0.55±0.50, P<0.001). MRS scores for hot flashes, sweating, heart discomfort, depressive mood, irritability, anxiety, physical and mental exhaustion, sexual problems, and bladder problems were significantly greater in women with a sleep disorder than in those without (all P<0.05). Women with a sleep disorder had a significantly higher total MRS score compared with those without (15.75±6.68 *vs*9.30±5.44, P*<*0.001), and this finding persisted with exclusion of the MRS sleep problems score (13.15±6.41 *vs*8.75±5.39, P*<*0.001).

### Relationship between the PSQI total score and the modified KI score according to menopausal status

Spearman's rank correlation coefficient indicated a strong positive correlation between the PSQI total score and the KI insomnia score for all of the patients (r_a_=0.791, P<0.001), women not in menopause (r_b_=0.834, P*<*0.001), and those who were menopausal (r_c_=0.762, P<0.001, Table 3). The PSQI total score for all of the patients was also very weakly (0-0.19) to weakly (0.20-0.39) positively correlated with the KI scores for sweating, hot flashes, nervousness, melancholia, fatigue, headache, heart palpitations, formication, and sexual complaints. In addition, the PSQI total score was significantly correlated with the KI score for hot flashes, headache, heart palpitations, and formication for women in menopause, but these correlations were not significant for women who were not menopausal. In contrast, the correlation between the PSQI total score and the KI score for sexual complaints and urinary tract infection was significant for women who were not menopausal, but not significant for women who were menopausal. Overall, the PSQI total score had a moderately positive correlation with the modified KI total score (r_a_=0.453, r_b_=0.474, and r_c_=0.430), but the strength of correlations became weaker if the KI insomnia score was excluded (r_a_=0.320, r_b_=0.352, and r_c_=0.286).

**Table:**
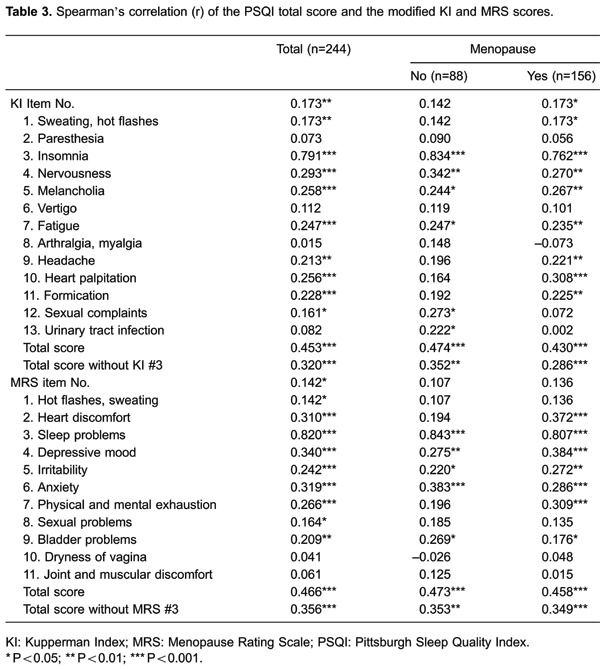

### Relationship between the PSQI total score and the MRS score according to menopausal status

Spearman's rank correlation coefficient indicated a strongly positive correlation between the PSQI total score and the MRS sleep problems score for all of the patients (r_a_=0.820, P<0.001), those not in menopause (r_b_=0.843, P<0.001), and those who were menopausal (r_c_=0.807, P<0.001, Table 3). The PSQI total score for all of the patients was very weakly (0-0.19) to weakly (0.20-0.39) positively correlated with the MRS scores for hot flashes, sweating, heart discomfort, depressive mood, irritability, anxiety, physical and mental exhaustion, sexual problems, and bladder problems. The PSQI total score was significantly correlated with heart discomfort and physical and mental exhaustion only for women who were menopausal. Overall, the PSQI total score was moderately positively correlated with the MRS total score (r_a_=0.466, r_b_=0.473, and r_c_=0.458), but the strength of correlations became weaker if the MRS sleep problems score was excluded (r_a_=0.356, r_b_=0.353, and r_c_=0.349).

## Discussion

Our study showed that correlations between the PSQI total score and insomnia and sleep problems scores were similar in menopausal and non-menopausal women. In addition, FSH and E2 levels were not associated with the presence of a sleep disorder in middle-aged women. These data do not support the hypothesis that menopause has a specific contribution to causing sleep problems.

The prevalence of sleep difficulties is increased in middle-aged and postmenopausal women (5,8,10,21). The reason for the association between sleep disorders and menopause is not clear (i.e., whether the increased incidence of sleep difficulties is associated with aging, or more specifically, with menopause). The Wisconsin Sleep Cohort Study used a probability sample of 589 premenopausal, perimenopausal, and postmenopausal women, and showed that menopause was not associated with diminished sleep quality as measured by polysomnography (22). This previous study also showed that although perimenopausal and postmenopausal women were less satisfied with their sleep relative to premenopausal women, menopause was not a strong predictor of specific sleep-disorder symptoms (22). The authors concluded that sleep abnormalities in midlife women should not be primarily attributed to menopause before ruling out underlying sleep disorders.

Some studies have reported that menopause has no effect on quality of sleep (23,24). However, some studies have shown that menopausal hot flashes and night sweats are associated with increased difficulty with sleep (25,26), and increased daytime sleepiness (23). The Study of Women's Health Across the Nation showed that vasomotor symptoms moderated the associations of anxiety with electroencephalographic sleep measures of sleep latency and sleep efficiency, and were associated with a longer duration of sleep (27). A recent population-based, 14-year, follow-up study of midlife women by Freeman et al. (28) reported that overall poor sleep did not increase around menopause and frequently occurred in the absence of hot flashes. The authors concluded that sleep difficulties in the perimenopausal period are not associated with ovarian decline.

Vasomotor symptoms are common during menopause, where hot flashes are the most frequent and are described as the most troublesome (7). Studies have generally shown that sleep disturbances during menopause may be associated with vasomotor symptoms (29). Joffe et al. (30) reported that quality of sleep, but not interruption of sleep, is worse in depressed than in nondepressed menopausal women with vasomotor symptoms. The authors also reported that the type of sleep disturbance observed in depressed participants was not consistent with the etiology of depression secondary to awakening associated with vasomotor symptoms. Vasomotor symptoms tend to positively correlate with a decline in estrogen levels during menopause. However, estrogen levels do not appear to be the only factor associated with vasomotor symptoms because these levels can be similar between asymptomatic and symptomatic women (31). Using the PSQI, Hung et al. (32) reported that menopause was associated with poor sleep quality in menopausal women without vasomotor symptoms.

The current study showed no difference in FSH or E2 levels in women with or without a sleep disorder. Other studies have provided varying results. Kalleinen et al. (33) studied older versus younger women and reported that sleep efficiency decreases with age and is not associated with decreasing estrogen levels. In contrast, other studies have shown greater difficulty in initiation and maintenance of sleep in postmenopausal woman than in premenopausal women, suggesting that sleep difficulties in middle-aged women are related to a decline in estrogen levels (34,35). Studies that examined the effect of estrogen supplementation on sleep disturbances in postmenopausal women have provided conflicting results. Some of these studies have indicated an improvement in sleeping and others have indicated no effect or a negative effect (10).

Depression and anxiety are common during menopause, and are both associated with sleep disturbances in otherwise healthy, non-menopausal women (36-38). Terauchi et al. (9) reported that insomnia in peri- and postmenopausal women was more closely associated with psychological than somatic symptoms. Their study also reported that difficulty initiating sleep was strongly correlated with anxiety, and non-restorative sleep was strongly correlated with depression. Correlation of depression and non-restorative sleep was also reported in a study by Ohayon et al. (39) that included more than 25,000 subjects from 7 European countries.

Some limitations of this study should be considered. Objective polysomnographic evidence of a sleep disorder was not obtained in the current study. We only evaluated sleep quality based on the PSQI. We did not evaluate patients for specific sleep disorders that are relatively common among middle-aged and menopausal women, such as obstructive sleep apnea, restless leg syndrome, periodic limb movement disorder, and fibromyalgia. Because this was not a cross-sectional study, the effect of menopause cannot be fully excluded. Furthermore, we did not consider a category of perimenopausal women, and some women who were classified as premenopausal may have been in the early stages of menopause instead. Pairing between groups was not performed. An objective laboratory measure, such as FSH levels, was not used to define patients who were postmenopausal. However, STRAW criteria do not require an FSH level to define menopause (16). Finally, further analysis of factors associated with good or poor sleepers in pre- and postmenopausal women would be of value. However, this analysis was beyond the scope of the current study.

Our study suggests that menopause *per se* does not appear to be important with respect to inducing sleep problems. Menopausal women with sleeping problems should be evaluated for other medical or psychological causes of poor sleep.

## References

[1] Chokroverty S (2009). Sleep disorders medicine: Basic science, technical
considerations and clinical aspects.

[2] Chokroverty S (2010). Overview of sleep & sleep disorders. Indian J Med Res.

[3] Léger D, Bayon V (2010). Societal costs of insomnia. Sleep Med Rev.

[4] Silber MH (2005). Clinical practice. Chronic insomnia. N Engl J Med.

[5] Ohayon MM (2002). Epidemiology of insomnia: what we know and what we still
need to learn. Sleep Med Rev.

[6] Krystal AD (2003). Insomnia in women. Clin Cornerstone.

[7] Avis NE, Stellato R, Crawford S, Bromberger J, Ganz P, Cain V (2001). Is there a menopausal syndrome? Menopausal status and
symptoms across racial/ethnic groups. Soc Sci Med.

[8] Kravitz HM, Zhao X, Bromberger JT, Gold EB, Hall MH, Matthews KA (2008). Sleep disturbance during the menopausal transition in a
multihethnic community sample of women. Sleep.

[9] Terauchi M, Hiramitsu S, Akiyoshi M, Owa Y, Kato K, Obayashi S (2012). Associations between anxiety, depression and insomnia in
peri- and post-menopausal women. Maturitas.

[10] Guidozzi F (2013). Sleep and sleep disorders in menopausal
women. Climacteric.

[11] Kupperman HS, Blatt MH, Wiesbader H, Filler W (1953). Comparative clinical evaluation of estrogenic
preparations by the menopausal and amenorrheal indices. J Clin Endocrinol Metab.

[12] Berlin Center for Epidemiology and Health Research (2014). MRS-the menopause rating scale.

[13] Schneider HP, Heinemann LA, Rosemeier HP, Potthoff P, Behre HM (2000). The Menopause Rating Scale (MRS): comparison with
Kupperman index and quality-of-life scale SF-36. Climacteric.

[14] Tao M, Shao H, Li C, Teng Y (2013). Correlation between the modified Kupperman Index and the
Menopause Rating Scale in Chinese women. Patient Prefer Adherence.

[15] Buysse DJ, Reynolds CF, Monk TH, Berman SR, Kupfer DJ (1989). The Pittsburgh Sleep Quality Index: a new instrument for
psychiatric practice and research. Psychiatry Res.

[16] Harlow SD, Gass M, Hall JE, Lobo R, Maki P, Rebar RW (2012). Executive summary of the Stages of Reproductive Aging
Workshop +10: addressing the unfinished agenda of staging reproductive
aging. Climacteric.

[17] Heinemann LA, Potthoff P, Schneider HP (2003). International versions of the Menopause Rating Scale
(MRS). Health Qual Life Outcomes.

[18] Tsai PS, Wang SY, Wang MY, Su CT, Yang TT, Huang CJ (2005). Psychometric evaluation of the Chinese version of the
Pittsburgh Sleep Quality Index (CPSQI) in primary insomnia and control
subjects. Qual Life Res.

[19] Wang XY, Yang HY, Nie GN, Wen ZH, Wu DR, Zhang CL (2008). [Study on the reliability and validity of the Chinese
Menopause Rating Scale (CMRS)]. Zhonghua Liu Xing Bing Xue Za Zhi.

[20] Heinemann LA, DoMinh T, Strelow F, Gerbsch S, Schnitker J, Schneider HP (2004). The Menopause Rating Scale (MRS) as outcome measure for
hormone treatment? A validation study. Health Qual Life Outcomes.

[21] Ohayon MM (2006). Severe hot flashes are associated with chronic
insomnia. Arch Intern Med.

[22] Young T, Rabago D, Zgierska A, Austin D, Laurel F (2003). Objective and subjective sleep quality in premenopausal,
perimenopausal, and postmenopausal women in the Wisconsin Sleep Cohort
Study. Sleep.

[23] Chasens ER, Twerski SR, Yang K, Umlauf MG (2010). Sleepiness and health in midlife women: results of the
National Sleep Foundation's 2007 Sleep in America poll. Behav Sleep Med.

[24] Chasens ER, Sereika SM, Weaver TE, Umlauf MG (2007). Daytime sleepiness, exercise, and physical function in
older adults. J Sleep Res.

[25] Kravitz HM, Zhao X, Bromberger JT, Gold EB, Hall MH, Matthews KA (2008). Sleep disturbance during the menopausal transition in a
multi-ethnic community sample of women. Sleep.

[26] Pien GW, Sammel MD, Freeman EW, Lin H, DeBlasis TL (2008). Predictors of sleep quality in women in the menopausal
transition. Sleep.

[27] Kravitz HM, Avery E, Sowers M, Bromberger JT, Owens JF, Matthews KA (2011). Relationships between menopausal and mood symptoms and
EEG sleep measures in a multi-ethnic sample of middle-aged women: the SWAN sleep
study. Sleep.

[28] Freeman EW, Sammel MD, Gross SA, Pien GW (2015). Poor sleep in relation to natural menopause: a
population-based 14-year follow-up of midlife women. Menopause.

[29] Freedman RR, Verster JC, Pandi-Perumal SR, Streiner DL (2008). Menopause, sleep, and quality of life. Sleep and quality of life in clinical medicine.

[30] Joffe H, Soares CN, Thurston RC, White DP, Cohen LS, Hall JE (2009). Depression is associated with worse objectively and
subjectively measured sleep, but not more frequent awakenings, in women with
vasomotor symptoms. Menopause.

[31] Freedman RR, Norton D, Woodward S, Cornelissen G (1995). Core body temperature and circadian rhythm of hot
flashes in menopausal women. J Clin Endocrinol Metab.

[32] Hung HC, Lu FH, Ou HY, Wu JS, Yang YC, Chang CJ (2014). Menopause is associated with self-reported poor sleep
quality in women without vasomotor symptoms. Menopause.

[33] Kalleinen N, Polo-Kantola P, Himanen SL, Alhola P, Joutsen A, Urrila AS (2008). Sleep and the menopause - do postmenopausal women
experience worse sleep than premenopausal women?. Menopause Int.

[34] Lukacs JL, Chilimigras JL, Cannon JR, Dormire SL, Reame NE (2004). Midlife women's responses to a hospital sleep challenge:
aging and menopause effects on sleep architecture. J Womens Health.

[35] Hachul de Campos H, Brandao LC, D'Almeida V, Grego BH, Bittencourt LR, Tufik S (2006). Sleep disturbances, oxidative stress and cardiovascular
risk parameters in postmenopausal women complaining of insomnia. Climacteric.

[36] Hollander LE, Freeman EW, Sammel MD, Berlin JA, Grisso JA, Battistini M (2001). Sleep quality, estradiol levels, and behavioral factors
in late reproductive age women. Obstet Gynecol.

[37] Cheng MH, Hsu CY, Wang SJ, Lee SJ, Wang PH, Fuh JL (2008). The relationship of self-reported sleep disturbance,
mood, and menopause in a community study. Menopause.

[38] Owens JF, Matthews KA (1998). Sleep disturbance in healthy middle-aged
women. Maturitas.

[39] Ohayon MM (2005). Prevalence and correlates of nonrestorative sleep
complaints. Arch Intern Med.

